# Outcomes of a Mobile Health Coaching Platform: 12-Week Results of a Single-Arm Longitudinal Study

**DOI:** 10.2196/mhealth.4933

**Published:** 2016-01-08

**Authors:** Steven Willey, James K Walsh

**Affiliations:** ^1^ St. Luke's Hospital Chesterfield, MO United States; ^2^ Senior Scientist Sleep Medicine and Research Center St. Luke's Hospital Chesterfield, MO United States

**Keywords:** weight loss, weight reduction program, obesity, aerobic exercise, resistance training, waist circumference, oxygen consumption

## Abstract

**Background:**

The number of mobile health coaching applications is expanding at a rapid rate. An application that uses a guiding intelligence to deliver an individualized structured program has the potential to provide a significant benefit. However, there are few studies of this approach that examine multiple clinical outcomes in a longitudinal manner.

**Objective:**

The objective of the study was to conduct a 12-week evaluation of participants using the YouPlus Health mobile coaching platform, specifically examining the effects on body weight, waist measurement, blood pressure, lipid profile, glycohemoglobin (A1C), and maximum volume of oxygen consumption (VO2 max).

**Methods:**

A quasi-experimental research design was used. This included a single-arm pre and post intervention assessment of outcomes. Participants underwent a 12-week intervention in which they received the entirety of the mobile health coaching program via an application on their mobile phones and were evaluated in the same physician’s office setting every two weeks. Data regarding app usage was continuously collected and maintained in a database.

**Results:**

10 subjects were enrolled in and completed the pilot study. The mean weight loss was 13.5 lbs. which represented 7.3% of baseline (*P*=.005). Mean waist circumference was reduced by 7.2 cm or 6.6% of baseline (*P*=.005). Both systolic (SBP) and diastolic (DBP) blood pressure measures were significantly lower after 12 weeks of intervention. Mean SBP fell 18.6 mmHg (*P*=.005) and mean DBP declined 6.4 mmHg (*P*=.005). VO2 max increased by an average of 3.13 ml/kg/min from baseline to study end (*P*=.005). From baseline to end-of-study HDL levels increased significantly by 4.0 mg/dL (*P*=.04) Total cholesterol, LDL, triglycerides, and glycohemoglobin (A1C) trended in the desired direction but did not meet statistical significance. All of the participants in the study completed the necessary in-app tutorials and also completed the in-app questions and received feedback. Every individual completed the appropriate amount of program levels necessary to give the specifics of the program, and the mean weekly app open rate ranged from 5.1 to 18.4.

**Conclusions:**

Users of the YouPlus Health mobile coaching platform experienced significant reductions in body weight, waist circumference, and both systolic and diastolic blood pressures, while attaining significant increases in HDL and VO2 Max.

## Introduction

Obesity is among the most crucial health issues facing the United States. Over 35.7 % of U.S. adults are obese, and another 33.1 % are overweight [1]. It is well established that obesity and body composition are associated with many chronic disease states, but it is also associated with diminished quality of life [2]. There is a tremendous economic cost as well, with an impact estimated to be upwards of 215 billion dollars annually [3].These realities have led to a tremendous effort to reduce these burdens and costs. This effort includes health coaching regarding appropriate diet and exercise behaviors. The traditional approach has used face to face interventions, but advances in technology provide ample opportunity to provide novel solutions via the internet and mobile phones. Currently, the available data shows promising trends in the success of these newer technologies [4]; however recent research regarding the choices available in app stores revealed a shortage of evidence-based apps. Out of 2400 apps initially searched, zero met guidelines for evidence-based aerobic physical activity and only 7 met evidence-based criteria for strength training [5].

Health coaching via digital means has its challenges; it must be doable and sustainable by the average person and ideally should include dietary and exercise guidelines that are supported by medical evidence. While the most widely downloaded apps in the digital-only space are counters, calculators, or trackers [6], an app that uses a guiding intelligence to provide an individualized structured program supported by scientific evidence could potentially more closely approximate face to face coaching and provide a substantial benefit. The YouPlus Health mobile coaching app was developed to provide this unique approach to digital coaching and was tested in this initial study.

As noted, the interventions of digital coaching need to be supported by medical evidence. While there are many dietary approaches, it is of note that higher protein diets have been associated with weight loss and maintenance of weight loss [7]. This effect is seen at quantities of at least 25-30 grams of protein per meal [7]. This effect is thought to be mediated through modulations in energy metabolism, appetite, and calorie intake via protein induced secretion of gastroentropancreatic hormones [8]. This also allows for carbohydrate consumption to be reduced. A meta-analysis of lower carbohydrate and higher protein diets has shown a favorable effect on body mass and composition independent of energy intake, including a 1.74 kg greater loss in body mass and a 1.29% greater decrease in body fat percentage [9]. Even small alterations in the pattern of dietary energy intake can produce significant results. One such example is a diet with an extra 5% more energy from protein and a 5% decreased energy from high glycemic index carbohydrates demonstrated weight reduction and also prevention of weight regain over the 6 month period after initial weight loss [10].

Combining a dietary approach with exercise has been shown to be superior to diet alone in inducing weight loss and metabolic changes [11]. National organizations have recommended that exercise include an aerobic, flexibility, and resistance component for many years [12].

Performing aerobic exercise increases cardiorespiratory fitness and has shown a linear association in decreasing early mortality when measuring exercise capacity in metabolic equivalents (METs) for both men and women [13-14], but there also appears to be a U-shaped association with all-cause mortality when looked at in the context of pace, quantity, and frequency of aerobic exercise [15]. For the best long term health outcomes, this needs to be considered in the design of a program in addition to its effect on weight.

The addition of resistance exercise has been shown to be more effective in inducing weight loss and improvement in body composition. While a hypocaloric balance is necessary for changing body composition, the effect of the imbalance regarding body compositional changes, and biomarkers including insulin levels and adipokines, is greater with the addition of resistance training [11,16]. A key element to effective resistance training is the proper prescription of the program variables. A program which uses progressive overload, variation, and specificity is essential to maximize the benefits associated with resistance training [17].

The purpose of this study was to evaluate a mobile phone app as a coaching tool for these evidence-based recommendations regarding diet and exercise behaviors, and to ascertain if app use was accompanied by positive measurable outcomes.

## Methods

### Research Design

A quasi-experimental research design was used. This included a single arm pre and post intervention assessment of body weight, waist measurement, blood pressure, lipid profile, glycohemoglobin (A1C), and maximum volume of oxygen consumption (VO_2max_). Data regarding app usage was continuously collected and maintained in a database. Participants were enrolled at no cost. They were not compensated for participation, but received the intervention and all related lab and VO2 testing at no cost.

### Participants

Individuals were recruited from the St. Louis area via print and internet advertisements for a mobile phone app-based health and fitness program. According to Flurry Analytics [18], 62% of health and fitness app users are females, and the 35-54 age group is over-indexed by 47%. Therefore, a study group approximating this demographic was desired. Respondents filled out an online questionnaire to determine eligibility for the study. Inclusion criteria were: 1) females between the ages of 30-50; 2) generally healthy with no significant medical history or use of prescription medications, and; 3) a BMI between 26.6 and 34. They were asked to be willing and able to exercise either at home or at a gym 3 times weekly. Participants attended an orientation meeting where consent forms were signed and the study process was explained. There was an opportunity to ask questions about the study. The app was downloaded during the orientation and the subjects completed the initial on-boarding assessment that ascertained their current diet and exercise habits during the meeting. Twelve subjects attended the orientation, and 10 then entered the study and subsequently completed the full 12 weeks as requested. Participants were Caucasian with an average age of 43.5 years (range 35-49) and an average BMI of 31.6 (range 27.2-36.4). All were non-smokers. All were either employed or served as a caretaker.

### Program

**Figure 1:**
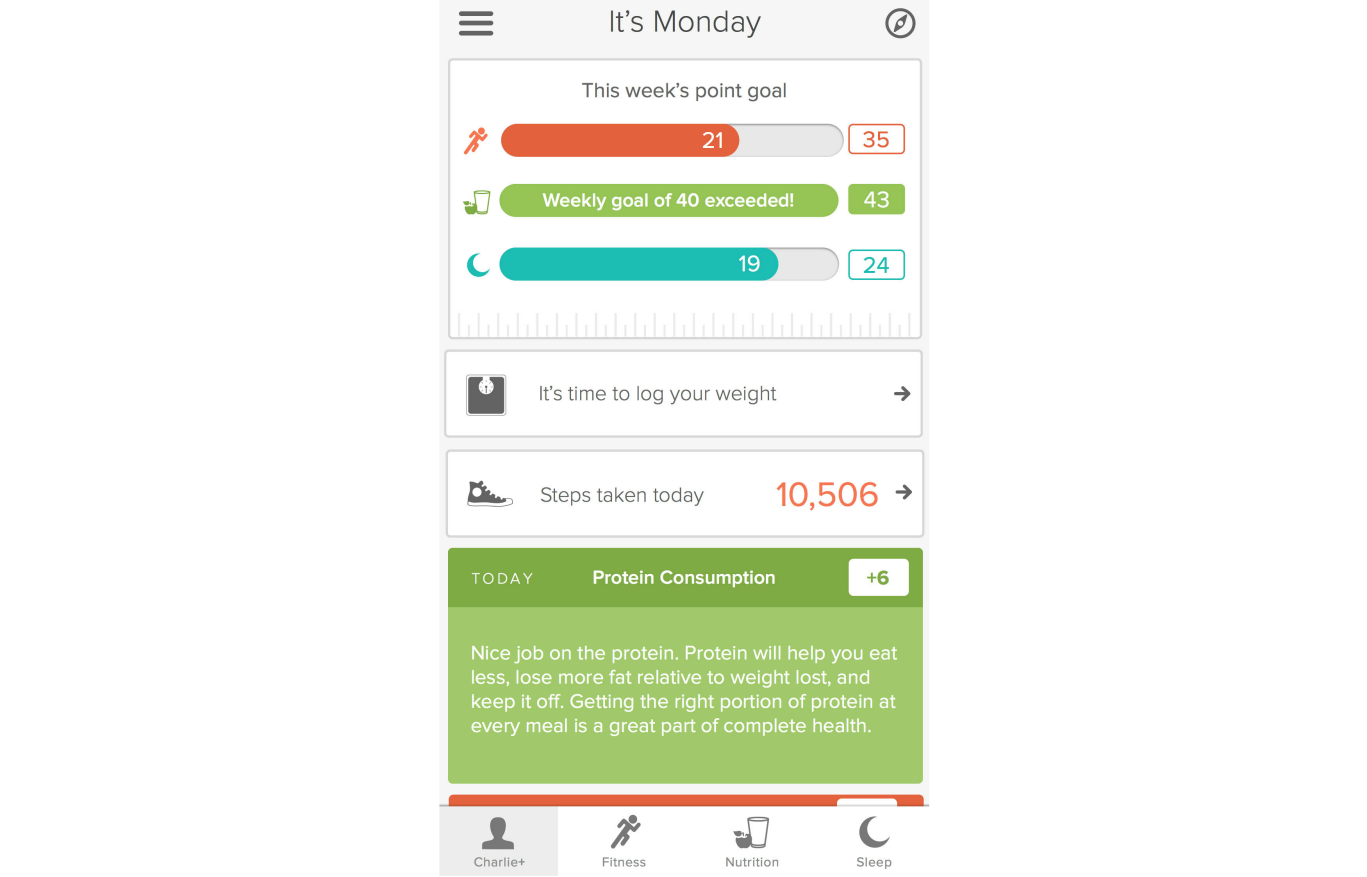
Sample profile screen and feedback.

**Figure 2:**
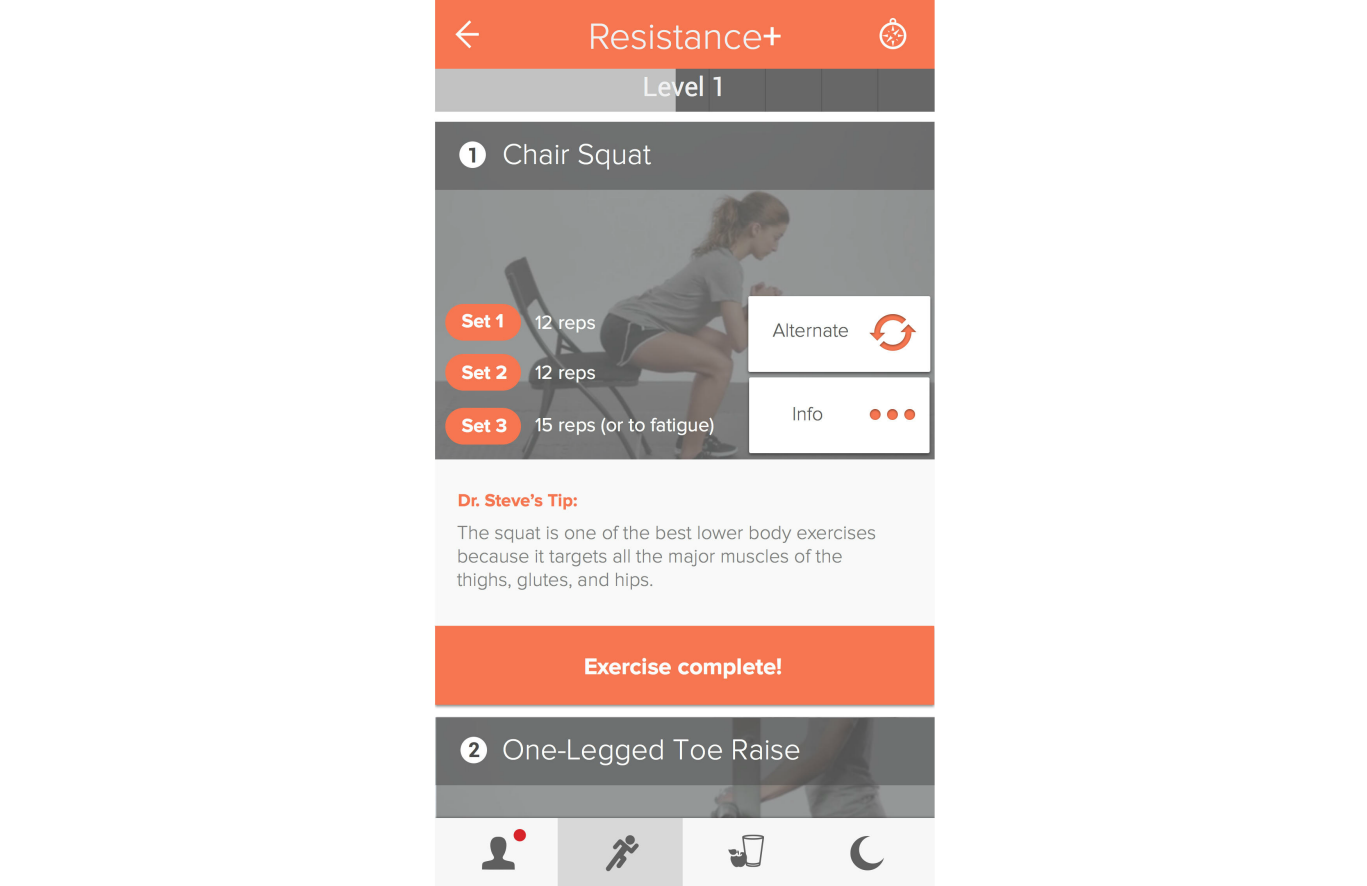
Sample exercise guidance screen.

The participants used the YouPlus Health mobile coaching platform to follow nutritional and exercise guidelines. There is a sleep tracking and improvement function of the platform, but it was not utilized during this study. Sample screen shots are demonstrated in Figures 1 and 2. Individuals received the entirety of the program via the app. Participants were asked to complete in-app tutorials, and as they advanced, each level of the program was only available to them by using the app.

The nutritional guidance on the platform uses a ratio-based approach to protein and carbohydrate intake based on the relative size of one component to the other. Unlimited intake of fruits and vegetables is encouraged. No calorie counting or recording was involved. There was in-app education provided on the nutritional concepts, as well as guidance by providing recipes when eating at home and menu recommendations when dining out. Participants received daily in-app questions regarding their nutritional behavior, and then received automated feedback and advice based on their answers to those questions. The number of questions given each individual could vary, depending on the response given. Similarly, there are multiple feedback options for each question and answer pairing which are presented based upon a proprietary algorithm.

The exercise program encompasses aerobic, resistance, and core exercises divided into three sessions that take under an hour. The specific variety, order, frequency, and progression of resistance and core exercises are given in a personal training format with instructions and video. This information was only available within the app. Completing 3 sessions each of core, cardiovascular, and resistance exercise completed an exercise level. The participants were asked to complete a level at least every 7-10 days. At the end of every level, in-app questions were asked about the exercise they had performed and how it was perceived. Based on their answers to those questions, automated feedback and advice was generated. Individuals could not move on in the program until the questions were answered. The number of questions given each individual could vary, depending on the number of exercise levels completed.

To more closely approximate real-world conditions, there were no specific instructions given for frequency of use of the app other than to follow the exercise program.

### Measures

Participants had a baseline measurement of weight, blood pressure, and waist circumference assessed in a physician’s office setting as well as baseline laboratory measurements of a lipid panel (total cholesterol, high density lipoprotein, low-density lipoprotein, triglycerides,) and a hemoglobin A1C which were performed at LabCorp in St. Louis, MO. At baseline and after 12-weeks VO_2max_ assessments were performed at Lifetime Fitness in Ellisville, MO.

Every two weeks the participants visited the physician’s office on the same day of the week and, while being observed by a medical assistant, were weighed on a single calibrated scale and had waist measurements and blood pressure measurements recorded through the end of the study. The protocol of the International Chair on Cardiometabolic Risk was followed for determining waist circumference. All blood pressure and waist assessments were conducted by a single physician using identical procedures throughout the study. After the last office visit, laboratory values and VO_2max_ were re-assessed as above.

App usage information regarding tutorial completion, levels completed, questions asked and answered, and total app opens was recorded throughout the study.

### Analysis

Paired-Sample Wilcoxon Signed Rank Tests were used to compare baseline and 12-week post-intervention values.  This non-parametric test was used because of the small sample size and non-normal distributions for some variables. All statistical comparisons were repeated with *t* tests with identical findings, but only the non-parametric results are presented below. Because this is the first study of this app, we had no prior data with which to calculate power, nor could we justify using published data from studies of other methods to influence the health outcomes of interest. Effect size estimates for the Wilcoxon tests were calculated and interpreted as proposed by Cohen [19]. Finally, Spearman rho and Pearson *r* correlation coefficients were calculated to examine potential associations of app use and outcome measures which showed significant group changes. Results were essentially the same with the parametric and non-parametric correlational methods, but only the non-parametric are presented below.

## Results

### App usage

Results are summarized in Table 1.

**Table 1:** Usage data for key parameters.

Participant	Completed nutrition tutorial	Completed fitness tutorial	Levels completed	Questions asked	Questions answered	Mean weekly opens
1	Yes	Yes	14	185	185	11.5
2	Yes	Yes	18	276	276	15.1
3	Yes	Yes	9	125	125	8.2
4	Yes	Yes	9	127	127	6.8
5	Yes	Yes	16	16	187	16.8
6	Yes	Yes	14	215	215	15.0
7	Yes	Yes	14	218	218	18.4
8	Yes	Yes	9	126	126	5.1
9	Yes	Yes	8	100	100	5.6
10	Yes	Yes	16	238	238	16.1

Table 2 contains mean and median baseline and 12-week post-intervention values for all outcome measures collected during the study, as well as the z-scores and 2-tail *P*-values for Wilcoxon analyses, and effect size estimates.

### Changes in Weight and Waist Measurement

Significant reductions in body weight and waist circumference were observed between baseline and 12-week measurements. Mean body weight declined 13.5 lbs. representing 7.3% of baseline (z=-2.805; *P*=.005). Mean waist circumference was reduced by 7.2 cm or 6.6% of baseline (z=-2.825; *P*=.005). Effect size was -.63 for both metrics suggesting a “large” magnitude of change.

### Changes in Blood Pressure and VO2max

For blood pressure, an average of the baseline and first 2-week visit was used to determine the beginning blood pressure, and an average of the 10-week and 12-week visits were used to determine the ending blood pressure measurements.

Both systolic (SBP) and diastolic (DBP) blood pressure measures were significantly lower after 12-weeks of intervention. Mean SBP fell 18.6 mmHg (z=-2.810 ; *P*=.005) and mean DBP declined 6.4 mmHg (z=-2.805; *P*=.005).

VO_2max_ increased by an average of 3.13 ml/kg/min from baseline to study end (z=2.803; *P*=.005).

Effect size was -.63 for both BP metrics and .63 for VO_2max_, all suggesting a “large” magnitude of change.

### Changes in Lipid and Glycohemoglobin Measures

From baseline to end-of-study HDL levels increased significantly by 4.0 mg/dL (z=2.044; *P*=.04) and there was a trend toward a reduction in total cholesterol of 10.5 mg/dL (z=-1.784; *P=*.07) and triglycerides of 27 mg/dL (z=-1.478, *P*=.07). There was a baseline to 12-week reduction in LDL of 9.1 mg/dl that not reach significance (z=-1.581; *P*=.11).

Hemoglobin A1C did not change significantly (**P*=.*10) although mean values moved in the desired direction (from 5.54 to 5.42%). All patients were in the normal range at baseline and at study end.

Effect size estimates for all lipid and glycohemoglobin measures indicated “medium” magnitudes of change.

**Table 2:** Baseline and 12-week values for all outcome measures.

Variable			Baseline	12-Week	Z score	*P* value	Effect size (*r*)
**Weight and waist measurement**							
	**Body weight (lbs)**						
		Mean (SD)	186.3 (15.0)	172.8 (17.0)			-.63
		Median	191.8	177.3	-2.805	.005	
	**Waist circumference (cm)**						
		Mean (SD)	108.3 (6.7)	101.1 (7.7)			-.63
		Median	112.0	104.5	-2.825	.005	
**Blood pressure and VO**_**2max**_							
	**Systolic BP (mmHg)**						
		Mean (SD)	136.4 (15.4)	117.8 (5.9)			-.63
		Median	132.0	118.5	-2.810	.005	
	**Diastolic BP (mmHg)**						
		Mean (SD)	80.9 (5.1)	74.5 (4.0)			-.63
		Median	80.0	74.0	-2.805	.005	
	**VO**_**2max**_						
		Mean (SD)	26.7 (2.9)	29.9 (3.8)			.63
		Median	27.6	29.9	2.803	.005	
	**Total cholesterol (mg/dL)**						
		Mean (SD)	191.2 (41.8)	180.7 (38.9)			-.4
		Median	175.5	172.5	-1.784	.07	
**Lipid and glycohemoglobin measures**							
	**HDL** ^a^ **(mg/dL)**						
		Mean (SD)	47.4 (10.5)	51.4 (8.6)			.46
		Median	44.5	51.5	2.044	.04	
	**LDL** ^b^ **(mg/dL)**						
		Mean (SD)	114.6 (36.7)	105.5 (30.2)			-.35
		Median	100.0	100.0	-1.581	.11	
	**Triglycerides (mg/dL)**						
		Mean (SD)	145.8 (54.4)	118.8 (65.7)			-.33
		Median	145.0	88.0	-1.478	.07	
	**Hemoglobin A1C (%)**						
		Mean (SD)	5.5 (0.3)	5.4 (0.3)			-.36
		Median	5.5	5.5	-1.630	.10	

^a^ High-density lipoprotein

^b^ Low-density lipoprotein

### Association of App Use and Outcome Measure Changes

Spearman rho correlation coefficients were calculated between two app use variables (levels of exercise achieved, and mean weekly app openings per week) and the difference score from baseline to 12-weeks for each of the outcome measures showing a significant group change (weight, waist, SBP, DBP, Trig, HDL and VO_2max_). None of the 14 correlations were statistically significant; however, all were in the desired direction (ie, more app use was associated with greater improvement in each health outcome). Actual rho values ranged from 0.109 to 0.442.

## Discussion

### Principal Findings

Results indicate that while completing a 12-week program utilizing the YouPlus Health mobile coaching platform subjects achieved significant reductions in body weight, waist circumference, systolic blood pressure and diastolic blood pressure, while achieving significant increases in VO_2max_ and HDL. All of these parameters achieved a large effect size except HDL, which had a medium effect size. Decreases in total cholesterol, LDL, triglycerides, and glycohemoglobin trended in the desired direction with a medium effect size, but did not meet statistical significance. Recognizing that the small sample size and restricted range in app use among participants limited the possibility of finding a statistically reliable association between app use and outcomes, each of the 14 associations examined was in the desired direction (participants with more app usage had greater improvements in health outcomes).

Individuals in the study received the entirety of the program via the app. All participants completed the in-app nutrition and fitness tutorials, and answered questions and received in-app feedback. Answering of questions was required to move on in the program, so the percentage of questions answered would be expected to be 100% if the participants were completing levels within the program. All participants completed the minimum number of exercise levels during the study. This would be 7 or more during the 72 day study period, because they were asked to complete a level a minimum of once every 10 days. Given that the usage data confirms that the individuals were using the app to obtain the education and program for the study, it appears the results were obtained secondary to utilization of the app. The lack of a control group cannot completely exclude the possibility that results were enhanced by participants visiting a physician’s office to obtain their measurements.

The mean weight loss of 7.3 % from the baseline measurement is generally considered clinically significant and exceeds the 5% the Food and Drug Administration (FDA) considers significant when evaluating the efficacy of weight loss medications [20]. Nearly all (9 out of 10) participants met or exceeded a loss of 5% of body weight during the study. It is notable that the mean weight loss of 13.5 lbs. exceeds the weight loss in the only prospective trial that examined consecutive members of Weight Watchers, which had a weight loss of 9.7 lbs. after 12 weeks for the 33 members (30%) who completed the program for that length of time [21].

The increase in VO_2max_ of 3.13 ml/kg/min is an increase of 0.9 METs. It has been shown that the Framingham Risk Score-adjusted mortality risk decreases by 17% for every 1 MET increase in exercise capacity at baseline [14]. The statistically significant increase in exercise capacity demonstrated in 12 weeks with this intervention would be expected to impact this outcome as well. Improvements in both systolic and diastolic blood pressure, a decrease in waist circumference, and an increase in HDL are also widely accepted as contributing to a reduced risk of disease.

### Limitations

Study limitations include a non-randomized, uncontrolled single-arm study design which precludes causal inference of the program to the outcomes. The study was limited by its small sample size, and only looked at females, so applicability of the findings to males is not known.

### Conclusions

Results of this study suggest that individuals using the YouPlus Health mobile coaching platform experienced significant reductions in body weight, waist circumference, systolic and diastolic blood pressures, and significant increases in HDL and VO_2max_ in 12 weeks.

Usage data indicates that the participants used the app to receive the education and specific program elements, and were able to understand and follow the program to the degree necessary to achieve significant results in the areas noted. Digital therapeutics in general have the potential to promote health and wellness with a small ongoing cost. These results indicate that the YouPlus Health mobile coaching platform specifically has this potential, and therefore further investigation using a randomized, parallel group, controlled design is warranted.

## Abbreviations

A1C: glycohemoglobin
DBP: diastolic blood pressure
FDA: Food and Drug Administration
HDL: high-density lipoprotein
LDL: low-density lipoprotein
MET: metabolic equivalents
SBP: systolic blood pressure
VO2max: maximum volume of oxygen consumption
